# How to implement minimally invasive duodenum-preserving total pancreatic head resection for patients with pancreatic head lesions: A retrospective study

**DOI:** 10.1097/MD.0000000000034608

**Published:** 2023-08-04

**Authors:** Xueqing Liu, Zixuan Hu, Xinbo Zhou, Jianzhang Qin, Zhongqiang Xing, Yunfei Liang, Jiayue Duan, Jia Liu, Jianhua Liu

**Affiliations:** a Hepatobiliary Surgery Department, Second Hospital of Hebei Medical University, Shijiazhuang, China; b Department of Radiology, Second Hospital of Hebei Medical University, Shijiazhuang, China.

**Keywords:** duodenum-preserving, laparoscopic, total pancreatic head resection

## Abstract

Laparoscopic duodenum-preserving pancreatic head resection (LDPPHR) has been widely reported. However, due to the challenges involved in performing total pancreatic head resection during operation, there are few studies reporting it. Between November 2016 and October 2022, we performed laparoscopic duodenum-preserving total pancreatic head resection (LDPPHRt) on 64 patients in the Department of Hepatobiliary Surgery, the Second Hospital of Hebei Medical University. Perioperative data of the patients such as age, gender, body mass index, operation time, blood loss, and postoperative hospital stay were collected and analyzed. This study included 40 women and 24 men aged 41.4 ± 15.7 years. All patients completed the surgery, and none of the patients underwent laparotomy. The average operation time was 275 (255, 310) min. The average postoperative hospital stay was 12 (10, 16) days. The rate of occurrence of pancreatic fistula was 10.9% (7/64), and that of the biliary fistula was 9.4% (6/64). One of the patients underwent cholangiojejunostomy 3 months after the operation due to painless jaundice and bile duct dilatation. By dissecting the space between the pancreatic head and duodenum, the posterior pancreatic duodenal arterial arch and the surface vascular network of the common bile duct (CBD) can be preserved. This ensures the success of LDPPHRt and avoids postoperative complications in the absence of intraoperative image guidance.

## 1. Introduction

The duodenum-preserving pancreatic head resection (DPPHR), first proposed by Professor Beger in 1972, is the standard surgical procedure for treating benign lesions of the pancreatic head.^[1]^ This operation ensures the integrity of the common bile duct (CBD) and duodenum and the hormone secreting hormone function of the duodenum while treating pancreatic head lesions.^[2]^ However, to avoid postoperative ischemic necrosis of the duodenum and CBD, part of the pancreatic tissue is left in the head of the pancreas to ensure blood supply, which also increases the risk of postoperative pancreatic fistula.

Previous study^[3]^ that investigated duodenal blood supply arteries showed that blood supply to duodenum and CBD could be guaranteed if the integrity of the posterior pancreaticoduodenal arterial arch was preserved. This study provided a theoretical basis for the complete resection of the pancreatic head. However, some surgeons^[4–6]^ worried that excessive removal of pancreatic tissue might damage the integrity of the vascular arch and thus chose to retain a small quantity of pancreatic tissue. To minimize the risk of pancreatic fistula, our center chose to remove the total pancreas from the lateral wall of the CBD and the medial side of the duodenal ring. Additionally, to avoid the large abdominal incision caused by open surgery and reduce postoperative hospital stay, all patients in this study underwent laparoscopic duodenum-preserving total pancreatic head resection (LDPPHRt). Previous studies^[7,8]^ have shown that laparoscopic surgery can reduce the incidence of complications compared with open surgery. By taking advantage of laparoscopic amplification, we can ensure the integrity of the vascular arch and reduce intraoperative blood loss. In this study we sought to investigate whether LDPPHRt could be safely performed without intraoperative fluorescence image guidance.

## 2. Material and methods

**Figure 1.**
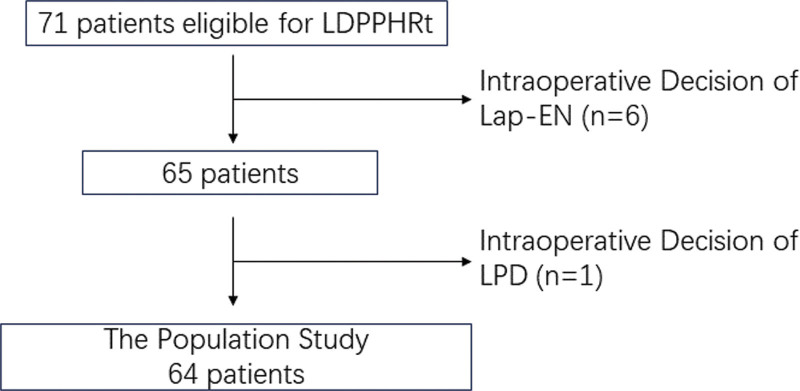
Patients selection for the study. Lap-EN = laparoscopic enucleation, LDPPHRt = laparoscopic duodenum-preserving total pancreatic head resection.

Between November 2016 and October 2022, we performed LDPPHRt on 64 patients (Fig. 1) in the Department of Hepatobiliary Surgery, the Second Hospital of Hebei Medical University. The perioperative data of patients such as age, gender, body mass index, operation time, blood loss, and duration of postoperative hospital stay were collected and analyzed. Written informed consent to participate was waived for its retrospective nature, and the patient data was deidentified to protect their privacy. The Ethics Committee of the Second Hospital of Hebei Medical University approved this study (No.2022-P031).

The following were the inclusion criteria: Preoperative CT or MRI showed benign lesions in the pancreatic head, with a large tumor bed or close association with the main pancreatic duct (MPD); presence of obvious symptoms, such as jaundice, abdominal pain, etc; This operation was performed for intraductal papillary mucinous neoplasia (IPMN) of the main duct type. The following were the exclusion criteria: patients who switched to laparotomy and patients with intraoperative freezing pathology with cancerous changes.

### 2.1. Surgical procedures

**Figure 2.**
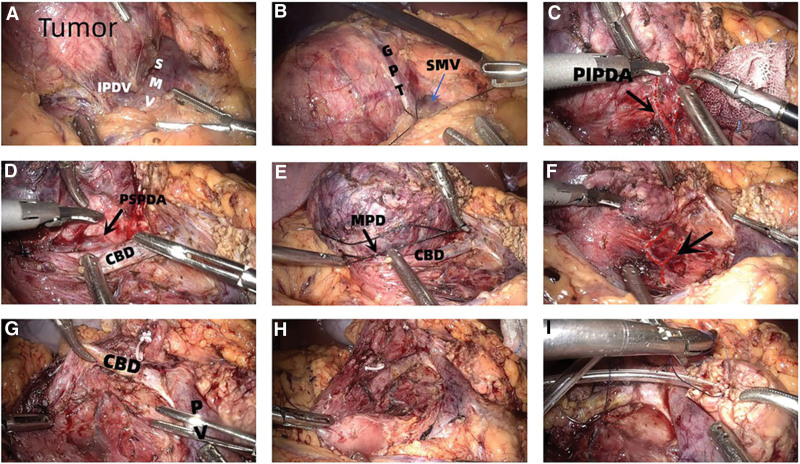
(A) The IPDV flows into the SMV. (B) Suture the GPT. (C) Close to pancreas when free the PIPDA. (D) The PSPDA can be seen behind the CBD. (E) Confirm ampulla and suture the MPD. (F) The posterior pancreatic fascia and the posterior pancreaticoduodenal arterial arch retain their integrity after the total head of the pancreas is removed. (G) There is no residual pancreatic tissue behind the CBD. (H) There was no residual pancreas in the medial duodenum and lateral wall of the CBD. (I) Hong PJ. CBD = common bile duct, GPT = gastric pancreatic trunk, IPDV = inferior pancreaticoduodenal vein, MPD = main pancreatic duct, PJ = pancreaticojejunostomy, PIPDA = posterior inferior pancreaticoduodenal artery, PSPDA = posterior superior pancreaticoduodenal artery, PV = portal vein, SMV = superior mesenteric vein.

After successful anesthesia, the patient was placed in a supine position with legs separated and head elevated. The 5 trocars were distributed in V-shape, with the center 1 cm below the umbilicus. The surgeon and the assistant stood on the right and left side of the patient, respectively, with the scope assistant standing between the legs. After a comprehensive abdominal exploration for potential abnormalities, an ultrasonic scalpel was used to disconnect the gastrocolic ligament to the right fusion fascia. Then the hepatic flexure of the colon was lowered to fully expose the pancreatic neck, head, and uncinate. Following this, the Kocher maneuver was not performed when freeing the duodenal peritoneum to protect the posterior pancreaticoduodenal vascular arch. The right gastroepiploic vein, right colonic vein, Henle trunk, and branches were isolated (Fig. 2A). Moreover, the superior mesenteric vein was exposed at the lower margin of the pancreas and the post-pancreatic neck tunnel was made. Then the pancreatic neck was cut off with an ultrasonic scalpel 1 cm away from the tumor, and the MPD was cut off with a scissor.

After the pancreatic neck was severed, the total head was gradually removed from the lower left to the front of upper right. The Henle trunk was then sutured, but in some patients, the gastric pancreatic trunk (Fig. 2B) was disconnected and the right colon vein was retained. The connective tissue between the pancreas and duodenum and posterior pancreaticoduodenal arterial arch was isolated against the pancreas (Fig. 2C–F). Then disconnect the branches of the arterial arch to the pancreas and protect the integrity of the posterior pancreaticoduodenal arterial arch. The ampulla and MPD near the duodenal papilla were carefully identified and then the MPD was sutured (Fig. 2E). Dissect the CBD upward, and find the CBD in combination with the pancreatic segment and the posterior segment of the duodenum if necessary. When the CBD was free, the surrounding pancreas was removed along the left and dorsal walls of the CBD (Fig. 2G). Therefore, the CBD and its surface vascular network could be fully preserved. Finally, the total pancreatic head was wholly resected (Fig. 2H), and the pancreatic stump was sent to the intraoperative frozen pathology. Based on the pathological results, decide whether to perform laparoscopic pancreatoduodenectomy (LPD) or not. During the operation, the Hem-o-lock should be reduced as much as possible, and sutures should be adopted to avoid bleeding after falling off of the clip due to improper operation. After the specimen was removed, the operative area was carefully irrigated, and then the CBD and duodenum were observed for blood disorders. Then the gauze was placed on the surface of the CBD to observe whether biliary fistula occurred.

For digestive tract reconstruction, the specimen was resected and one end of a pancreatic drainage tube with appropriate diameter and length was placed into the MPD by 4 cm. The 4-0 absorbable suture was used to suture the pancreas, the MPD and the pancreatic drainage tube, and the knot was fixed beside the drainage tube (Fig. 2I). Then a hole was drilled 10cm away from the stump of jejunum or the posterior wall of the stomach, and the diameter of the hole was like that of the pancreatic drainage tube. Then the purse-string suturing was performed around the hole with a 4-0 absorbable suture without tying knots. After placing the other end of the pancreatic drainage tube into the intestinal cavity or stomach cavity, the purse-string suture was tightened and tied for fixation. Finally, the ventral and dorsal sides of the pancreas and the serous layer of the digestive tract were continuously sutured with absorbable sutures. When suturing, the sutures did not penetrate the entire layer of the digestive tract wall, and thus the reconstruction of the digestive tract was completed.

Finally, the surgical area was rinsed and drainage tubes were placed at the pancreatic anastomosis and the original pancreatic head area. Then it was again observed whether there was ischemic necrosis in the CBD and duodenum.

### 2.2. Follow-up and statistical analyses

In this study, we adopted telephone or outpatient follow-up and asked the patients to check the upper abdomen Contrast Enhanced Computed Tomography and tumor markers 3 months after discharge. SPSS 26.0 software were used to analyze data. The normal distribution measurement data is expressed as (*x̄*±*s*), and the skewed distribution measurement data is expressed as [M (Q1, Q3)].

## 3. Results

The preoperative data of patients in this study are shown in Table 1. This study included 40 females and 24 males, aged 41.4 ± 15.7 years, with a body mass index of 22.9 (21.5, 24.5) kg/m^2^.

**Table 1:** Preoperative characteristics.

Characteristics	Value
Sex	
Male: female	24:40
Age, yr	41.4 ± 15.7
BMI, kg/m^2^	22.9 (21.5, 24.5)
Comorbidity	
Post-appendectomy	1
Hypertension	7
Diabetes	4
Cerebral infarction	1

BMI = body mass index.

Intraoperative data are presented in Table 2. All patients completed the operation without conversion to laparotomy. The average operation time was 275 (255, 310) min, the average intraoperative blood loss was 200 (100, 300) mL, and 5 patients required blood transfusion.

**Table 2:** Intraoperative indicators.

Indicators	Value
Time, min	275 (255, 310)
Blood loss, mL	200 (100, 300)
Transfusion requirement (n, %)	5, 7.8%
Negative margin	64
Conversion	0

Table 3 lists the indicators of postoperative patient recovery. There were no perioperative deaths and duodenal ischemia in this study. The average postoperative hospital stay was 12 (10,16) days, and the average postoperative gastrointestinal function recovery time was 48 (24–96) hours. The median longest tumor diameter was 6.5cm, ranging from 1.2 to 11 cm. The pancreatic fistula rate was 10.9% (7/64). Five patients with grade B fistula improved after adequate drainage and somatostatin treatment. Two patients with grade C fistula underwent laparoscopic hemostasis due to abdominal hemorrhage. One case with pancreatic wound hemorrhage showed improvement after conservative treatment. The biliary fistula rate was 9.4% (6/64), of which 3 cases were cured after implantation of a choledochal stent, and 3 cases showed improvement after complete drainage. The postoperative pathologic diagnosis included 22 cases of solid pseudopapillary tumors, 15 cases of IPMN, 9 cases of serous cystic cystadenoma, 8 cases of mucinous cystadenoma, 7 cases of pancreatic neuroendocrine tumors, 1 case of the true pancreatic cyst, 1 case of the calcified nodule, and 1 case of hemangioma.

**Table 3:** Postoperative characteristics.

Indicators	Value
Complication	
Biliary fistula (n, %)	6, 9.4%
Pancreatic fistula (n, %)	
Biochemical fistula	6, 9.4%
Grade B	5, 7.8%
Grade C	2, 3.1%
Abdominal bleeding (n, %)	1, 1.6%
Duodenal necrosis (n, %)	0
CBD stenosis (n, %)	1, 1.6%
Recovery of gastrointestinal function, h	48(range, 24–96)
Postoperative hospital stays, d	12(10, 16)
90-d mortality	0
R0	64
Pathology	
SPT	22
IPMN	15
Serous cystadenoma	9
Mucinous cystadenoma	8
PNET	7
TPC, calcified nodule, hemangioma	1:1:1
Tumor size, cm	6.5 (range, 1.2–11)

CBD = common bile duct, IPMN = intraductal papillary mucinous neoplasia, PNET = pancreatic neuroendocrine tumors, SPT = solid pseudopapillary tumors, TPC = true pancreatic cyst.

The long-term complications were as follows: 1 patient was diagnosed with biliary fistula 12 days after the operation, which improved after choledochal stent placement. However, cholangiojejunostomy was performed 3 months after the operation due to painless jaundice and bile duct dilatation. Two patients developed diarrhea with weight loss after LDPPHRt and improved after pancreatic enzyme supplementation.

## 4. Discussion

Traditional DPPHR does not remove the stomach, duodenum, and CBD, avoiding the complications such as cholangitis-intestinal anastomotic stricture, and internal and external secretion dysfunction after digestive tract reconstruction. The key to success of the DPPHR is to ensure an intact blood supply to the duodenum and CBD and avoid ischemic necrosis. To this end, Professor Beger^[9]^ chose to preserve the pancreas with a thickness of about 0.5 cm behind the CBD and at the medial margin of the duodenum. However, secretion from the residual pancreas can significantly increase the probability of postoperative pancreatic fistula. Therefore, some scholars^[8]^ tried to uncover the CBD and completely remove the pancreas inside the duodenal ring to ensure the integrity of the posterior pancreaticoduodenal vascular arch.

DPPHR was initially used clinically to treat inflammatory masses in the pancreas caused by chronic pancreatitis (CP).^[10]^ Later, it was extended to benign and low-grade malignancies such as IPMN, solid pseudopapillary tumors, and pancreatic neuroendocrine tumors. In the past, the classical surgical method for treating these diseases was pancreaticoduodenectomy (PD).^[11]^ However, PD involves a significant number of excised organs and complex digestive tract reconstruction. DPPHR is an organ-preserving surgery with minor surgical trauma. It has obvious advantages over classical PD in shortening hospital stay, rapid recovery and preserving the internal and external secretory function of pancreas.^[12]^ Moreover, it improves the long-term prognosis of patients in comparison to PD.^[13]^ However, in patients with CP, the inflammatory reaction of the tissues around the pancreatic head results in heavy tissue adhesion and unclear anatomical level. To avoid long operation time and intraoperative accidental injury, our department temporarily did not take CP as the surgical indication of LDPPHRt. In addition, although the pathological diagnosis could be confirmed by preoperative puncture, we did not routinely perform preoperative endoscopic ultrasonography-guided fine needle aspiration, as the puncture could cause inflammation in the pancreatic head and thus complicate the surgery.

The distal CBD and the Vater ampulla are mainly supplied by the posterior superior pancreaticoduodenal artery and its branches. Therefore, preserving the posterior arterial arch during the operation is necessary to ensure the blood supply.^[14]^ In this study, our department performed LDPPHRt from left to right and from bottom to top. Preservation of the posterior arterial arch was mainly achieved by operating close to the pancreas and disconnecting the branch vessels to the pancreas without overexposure of the posterior arterial arch and making a Kocher maneuver. Although Professor Wang^[15]^ believed that there were no perforating vessels from the retroperitoneum to supply the duodenum, the arterial branches supplying the duodenum were accompanied by veins. Even if the duodenal peritoneum were incised, the intestinal wall blood return would not be affected. However, avoiding Kocher maneuver is still the consensus of most scholars.^[8,16,17]^ In the case of intraoperative bleeding, we first chose compression to stop bleeding to avoid blood vessel damage due to unclear vision.

According to the position relationship between the CBD and pancreas, CBD is divided into the dorsal and intrapancreatic types. Based on the clinical experience of our department in protecting CBD without intraoperative image guidance, the dorsal CBD of the pancreas was recognized behind the pancreas and the pancreas was removed from bottom to top. However, the intrapancreatic CBD should be identified behind gastroduodenal arteria and to the right of the portal vein. Then it was dissociated in the upper and lower directions. After resecting of the pancreatic head, the residual pancreas on the medial edge of the duodenum and CBD was further removed. Finally, the blood supply of duodenum and CBD was observed. In the absence of apparent ischemia or biliary fistula, the digestive tract could be reconstructed. Some scholars^[5,17,18]^ have innovatively applied the laparoscopic fluorescence imaging system, enabling the visualization and preservation of the CBD and vessels supplying the duodenum, and the timely identification of biliary fistula. However, our center used a 3D laparoscopic lens during surgery, while the fluorescent imaging system needed a 2D lens. Considering that intraoperative lens conversion could affect the flow of the whole operation, intraoperative fluorescence image guidance was not used in this study.

**Figure 3.**
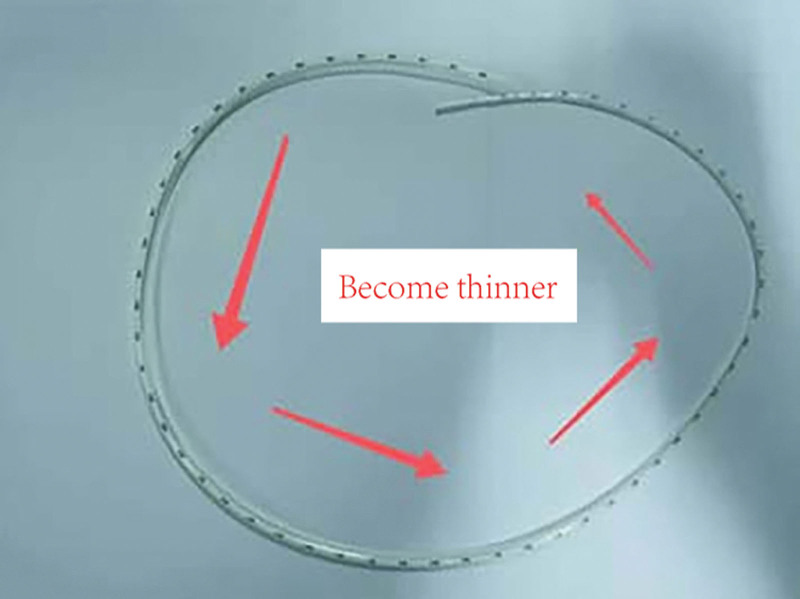
Variable diameter measurable pancreatic duct. The pipe diameter gradually becomes thinner along the arrow direction.

Regarding digestive tract reconstruction methods, Hong PJ^[19]^ was selected at the initial stage. Later, the modified pancreaticogastrostomy based on Hong PJ was performed. The “Variable diameter measurable pancreatic duct” invented by Professor Liu^[20]^ was used as the pancreatic duct support tube for both anastomosis methods (Fig. 3). During the operation, the corresponding part of the pancreatic duct was excised according to the different MPD diameters, which fit perfectly fitted without pancreatic fluid leakage after insertion into the MPD.

The effect of total pancreatic head resection on reducing pancreatic fistula is significant. In 295 patients who underwent subtotal pancreatic head resection reported by Beger in 2016, the pancreatic fistula rate, excluding biochemical fistula, was 13.6%.^[21]^ But the pancreatic fistula rate of 64 patients who underwent total pancreatic head resection in our center was lower (10.9%). In the later stage, to further reduce the rate of pancreatic fistula, 11 patients were selected for modified pancreaticogastrostomy anastomosis, and no pancreatic fistula occurred in these patients. In our center, pancreatic fistula is managed by thoroughly draining to avoid abdominal infection and bleeding. In this study, 5 patients with grade B pancreatic fistula were discharged after conservative treatment. CBD stent placement is the preferred treatment method in our center besides adequate drainage for treating biliary fistula. Three patients with biliary fistula improved after the placement of the choledochal stent. And only 1 patient was treated with choledochal stenosis 3 months after the operation. This also demonstrated the safety and improved long-term prognosis of LDPPHRt performed in our department.

The current study has some limitations. Although clinical data of 64 patients were included in this study, there was no corresponding control group. This was because our center had not previously performed secondary pancreatectomy. Secondly, this study included digital surgeons, and due to varying degrees of mastery of surgery, there are certain differences in surgical indicators.

## 5. Conclusion

LDPPHRt is a safe and reliable surgical method that can be successfully performed without intraoperative image guidance. By dissecting the space between the pancreatic head and duodenum, the posterior pancreatic duodenal arterial arch and the surface vascular network of the CBD can be preserved. This ensures the success of LDPPHRt and prevents postoperative complications.

## Author contributions

**Data curation:** Yunfei Liang.

**Formal analysis:** Zixuan Hu.

**Investigation:** Jianzhang Qin, Zhongqiang Xing.

**Methodology:** Jiayue Duan.

**Project administration:** Xueqing Liu.

**Software:** Xinbo Zhou, Jia Liu.

**Writing – original draft:** Zixuan Hu.

**Writing – review & editing:** Xueqing Liu, Jianhua Liu.

## References

[1] Beger HG Rau BM Gansauge F. Duodenum-preserving subtotal and total pancreatic head resections for inflammatory and cystic neoplastic lesions of the pancreas. J Gastrointest Surg. 2008;12:1127–32. 18299945 10.1007/s11605-008-0472-4

[2] Ito K. Duodenum preservation in pancreatic head resection to maintain pancreatic exocrine function (determined by pancreatic function diagnostant test and cholecystokinin secretion). J Hepatobiliary Pancreat Surg. 2005;12:123–8. 15868075 10.1007/s00534-004-0954-z

[3] Takada T Yasuda H Uchiyama K. Duodenum-preserving pancreatoduodenostomy. A new technique for complete excision of the head of the pancreas with preservation of biliary and alimentary integrity. Hepatogastroenterology. 1993;40:356–9. 8406305

[4] Chai W Zhang Z Lei B. Analysis of surgical complications after laparoscopic duodenum-preserving pancreatic head resection for noncancerous lesions. Chin J Gen Surg. 2022;37:443–6.

[5] Cai Y Zheng Z Gao P. Laparoscopic duodenum-preserving total pancreatic head resection using real-time indocyanine green fluorescence imaging. Surg Endosc. 2021;35:1355–61. 32221750 10.1007/s00464-020-07515-6

[6] Zhou J Zhou Y Mou Y. Laparoscopic duodenum-preserving pancreatic head resection: a case report. Medicine (Baltim). 2016;95:e4442. 10.1097/MD.0000000000004442 PMC4985314 27512859

[7] Beger HG Nakao A Mayer B. Duodenum-preserving total and partial pancreatic head resection for benign tumors--systematic review and meta-analysis. Pancreatology. 2015;15:167–78. 25732271 10.1016/j.pan.2015.01.009

[8] Cao J Li GL Wei JX. Laparoscopic duodenum-preserving total pancreatic head resection: a novel surgical approach for benign or low-grade malignant tumors. Surg Endosc. 2019;33:633–8. 30456509 10.1007/s00464-018-6488-2

[9] Beger HG Büchler M Bittner RR. Duodenum-preserving resection of the head of the pancreas in severe chronic pancreatitis. Early and late results. Ann Surg. 1989;209:273–8. 2923514 10.1097/00000658-198903000-00004 PMC1493931

[10] Beger HG Krautzberger W Bittner R. Duodenum-preserving resection of the head of the pancreas in patients with severe chronic pancreatitis. Surgery. 1985;97:467–73. 3983823

[11] Stauffer JA Coppola A Villacreses D. Laparoscopic versus open pancreaticoduodenectomy for pancreatic adenocarcinoma: long-term results at a single institution. Surg Endosc. 2017;31:2233–41. 27604369 10.1007/s00464-016-5222-1

[12] Diener MK Rahbari NN Fischer L. Duodenum-preserving pancreatic head resection versus pancreatoduodenectomy for surgical treatment of chronic pancreatitis: a systematic review and meta-analysis. Ann Surg. 2008;247:950–61. 18520222 10.1097/SLA.0b013e3181724ee7

[13] Qin H Yang S Yang W. Duodenum-preserving pancreas head resection in the treatment of pediatric benign and low-grade malignant pancreatic tumors. HPB (Oxford). 2020;22:306–11. 31409540 10.1016/j.hpb.2019.06.009

[14] Kim SW Kim KH Jang JY. Practical guidelines for the preservation of the pancreaticoduodenal arteries during duodenum-preserving resection of the head of the pancreas: clinical experience and a study using resected specimens from pancreaticoduodenectomy. Hepatogastroenterology. 2001;48:264–9. 11268981

[15] Xie B Wang H. Key points and indications of duodenum-preserving pancreatic head resection. J Hepatobiliary Surg. 2014;22:5–7.

[16] Peng CH Shen BY Deng XX. Early experience for the robotic duodenum-preserving pancreatic head resection. World J Surg. 2012;36:1136–41. 22415757 10.1007/s00268-012-1503-6

[17] Hong D Cheng J Wu W. How to perform total laparoscopic duodenum-preserving pancreatic head resection safely and efficiently with innovative techniques. Ann Surg Oncol. 2021;28:3209–16. 33123857 10.1245/s10434-020-09233-8

[18] Chen S Gao P Cai H. Indocyanine green-enhanced fluorescence in laparoscopic duodenum-preserving pancreatic head resection: technique with video. Ann Surg Oncol. 2020;27:3926–7. 32266569 10.1245/s10434-020-08360-6

[19] Hong D Liu Y Zhang Y. The role of Hong’s single-stitch duct to mucosa pancreaticojejunostomy in laparoscopic pancreaticoduodenectomy. Chin J Surg. 2017;55:136–40. 28162214 10.3760/cma.j.issn.0529-5815.2017.02.012

[20] Li Q Li A Xing Z. Clinical application of “variable diameter measurable pancreatic duct” in laparoscopic pancreaticoduodenectomy. Chin J Hepatobiliary Surg. 2021;27:411–4.

[21] Beger HG Mayer B Rau BM. Parenchyma-sparing, limited pancreatic head resection for benign tumors and low-risk periampullary cancer--a systematic review. J Gastrointest Surg. 2016;20:206–17. 26525207 10.1007/s11605-015-2981-2

